# Microbial iron management mechanisms in extremely acidic environments: comparative genomics evidence for diversity and versatility

**DOI:** 10.1186/1471-2180-8-203

**Published:** 2008-11-24

**Authors:** Héctor Osorio, Verónica Martínez, Pamela A Nieto, David S Holmes, Raquel Quatrini

**Affiliations:** 1 Center for Bioinformatics and Genome Biology, Fundación Ciencia para la Vida, MIFAB, Santiago, Chile; 2 Depto. de Ciencias Biologicas, Facultad de Ciencias de la Salud, Universidad Andres Bello, Santiago, Chile

## Abstract

**Background:**

Iron is an essential nutrient but can be toxic at high intracellular concentrations and organisms have evolved tightly regulated mechanisms for iron uptake and homeostasis. Information on iron management mechanisms is available for organisms living at circumneutral pH. However, very little is known about how acidophilic bacteria, especially those used for industrial copper bioleaching, cope with environmental iron loads that can be 10^18 ^times the concentration found in pH neutral environments. This study was motivated by the need to fill this lacuna in knowledge. An understanding of how microorganisms thrive in acidic ecosystems with high iron loads requires a comprehensive investigation of the strategies to acquire iron and to coordinate this acquisition with utilization, storage and oxidation of iron through metal responsive regulation. *In silico *prediction of iron management genes and Fur regulation was carried out for three Acidithiobacilli: *Acidithiobacillus ferrooxidans *(iron and sulfur oxidizer) *A. thiooxidans *and *A. caldus *(sulfur oxidizers) that can live between pH 1 and pH 5 and for three strict iron oxidizers of the *Leptospirillum *genus that live at pH 1 or below.

**Results:**

Acidithiobacilli have predicted FeoB-like Fe(II) and Nramp-like Fe(II)-Mn(II) transporters. They also have 14 different TonB dependent ferri-siderophore transporters of diverse siderophore affinity, although they do not produce classical siderophores. Instead they have predicted novel mechanisms for dicitrate synthesis and possibly also for phosphate-chelation mediated iron uptake. It is hypothesized that the unexpectedly large number and diversity of Fe(III)-uptake systems confers versatility to this group of acidophiles, especially in higher pH environments (pH 4–5) where soluble iron may not be abundant. In contrast, Leptospirilla have only a FtrI-Fet3P-like permease and three TonB dependent ferri-dicitrate siderophore systems. This paucity of iron uptake systems could reflect their obligatory occupation of extremely low pH environments where high concentrations of soluble iron may always be available and were oxidized sulfur species might not compromise iron speciation dynamics. Presence of bacterioferritin in the Acidithiobacilli, polyphosphate accumulation functions and variants of FieF-like diffusion facilitators in both Acidithiobacilli and Leptospirilla, indicate that they may remove or store iron under conditions of variable availability. In addition, the Fe(II)-oxidizing capacity of both *A. ferrooxidans *and Leptospirilla could itself be a way to evade iron stress imposed by readily available Fe(II) ions at low pH. Fur regulatory sites have been predicted for a number of gene clusters including iron related and non-iron related functions in both the Acidithiobacilli and Leptospirilla, laying the foundation for the future discovery of iron regulated and iron-phosphate coordinated regulatory control circuits.

**Conclusion:**

*In silico *analyses of the genomes of acidophilic bacteria are beginning to tease apart the mechanisms that mediate iron uptake and homeostasis in low pH environments. Initial models pinpoint significant differences in abundance and diversity of iron management mechanisms between Leptospirilla and Acidithiobacilli, and begin to reveal how these two groups respond to iron cycling and iron fluctuations in naturally acidic environments and in industrial operations. Niche partitions and ecological successions between acidophilic microorganisms may be partially explained by these observed differences. Models derived from these analyses pave the way for improved hypothesis testing and well directed experimental investigation. In addition, aspects of these models should challenge investigators to evaluate alternative iron management strategies in non-acidophilic model organisms.

## Background

Natural geomicrobiological processes and industrial operations, such as coal mining and bioleaching, can generate extremely acidic environments (pH 1) in which insoluble metal sulfides are converted into water-soluble metal sulfates that include extraordinarily high concentrations of soluble iron. These concentrations can reach values as high as 160 g/L, about 10^18 ^higher than typically found in circumneutral environments.

In oxygen saturated environments at neutral pH, Fe(II) is readily oxidized to Fe(III) [1]. Thus, iron predominantly occurs in the ferric form as poorly soluble iron hydroxides (as low as 10–18 M at pH 7.0), rendering it basically unavailable for biological systems [2]. In contrast, under acidic conditions Fe(II) persists for long periods of time even in the presence of atmospheric oxygen [1] and aerobic acidophiles have to cope with the highest levels of soluble iron in nature and the threat it imposes via its reaction with oxygen, generating free radicals that damage macromolecules and cause cell death [3].

A number of biological processes have evolved to deal with metal-induced threats to life including the ability to transform [4], sequester intra- or extracellularly [5], exclude [6] or remove [7] potentially toxic ions. In the case of metals that are also essential micronutrients, such as iron, concentration-dependent toxicity is coped with by carefully balancing influx and efflux, preserving intracellular metal homeostasis [8]. In the case of iron, the accepted view is that cells respond to iron-dependent oxidative stress by down-regulating iron uptake, promoting its utilization and depositing surplus iron in storage proteins, as well as by mitigating the effects of emerging reactive oxygen species [9]. Most prokaryotes coordinate and regulate these processes by means of the ferric uptake regulator Fur, which serves as a global regulator of gene expression by responding to changes in iron availability.

In contrast to the wealth of information available for neutrophiles, many unanswered questions remain regarding the nature and ecological distribution of the genetic determinants underlying iron management mechanisms in acidophiles. This understanding is essential for generating a comprehensive description of the ecophysiology of these microorganisms and for understanding their contributions to the cycling of iron in pristine environments. In addition, microbial cycling of iron in acidic conditions is important for understanding bioleaching of ores and the development of remediation techniques for sites affected by acid mine drainage or contaminated with metals from industrial wastes including coal heaps [10]. For example, ecological effects of the Fe(III) tolerance have been suggested to explain the dominance of Leptospirilla over *Acidithiobacillus ferrooxidans *[11] and of *Sulfobacillus acidophilus *over *Acidimicrobium ferrooxidans *[12] in mixed cultures oxidizing Fe(II), even if the underlying mechanisms were not elucidated. An understanding of how microorganisms thrive in acidic ecosystems with high iron loads requires a comprehensive investigation of the strategies to acquire iron and to coordinate this acquisition with utilization, storage and oxidation of iron through metal responsive regulation. It is also necessary to understand how oxidative stress caused by iron overload is mitigated.

In this work, aspects of iron homeostasis responses are reported for some of the major contributors to microbial bioleaching through multiple *in silico *genomic comparisons of currently available completed [13] and draft genome sequences [14-16], contrasting them with what is already known in *A. ferrooxidans *[17,18]. Using bioinformatics and comparative genomic strategies, models have been constructed for Acidithiobacilli and Leptospirilla species for a) the genetic determinants of iron management, b) the Fur-dependent genetic regulatory network and c) the gene complements and relevant aspects of the iron homeostasis response.

## Results

### Ferrous Iron Transporter Profiles

**Figure 1:**
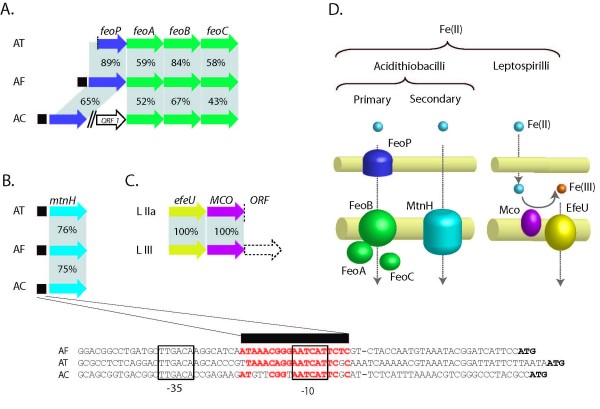
**Predicted ferrous iron transporter gene organization and function in Acidithiobacilli and Leptospirilla.** A) FeoPABC system, B) NRAMP-family transporter MntH with the DNA sequences of their predicted Fur boxes, C) EfeU-MCO system, D) Model for ferrous iron transport. ■: predicted Fur box. AT: *A. thiooxidans*; AF: *A. ferrooxidans*; AC: *A. caldus*; LIIa: *Leptospirillum *sp. Group II UBA; LIIb: *Leptospirillum *sp. Group II 5-way GC; LIII: *Leptospirillum *sp. Group III 5-way GC.

The major route for Fe(II) acquisition in neutrophilic bacteria, including pathogens, is via the FeoB uptake system [19]. A candidate gene with similarity to *feoB *with an associated upstream, experimentally validated, Fur regulatory binding site (Fur box) has been described previously in *A. ferrooxidans *[18,20]. In *A. ferrooxidans*, *feoB *is conserved in the gene context *feoABC *that is typical of the Fe(II) uptake system described in other bacteria; however, in this case, it is preceded by a putative dedicated permease-encoding gene, *feoP*. Genes with similarity to *feoPABC *were also predicted in *Acidithiobacillus thiooxidans *and *Acidithiobacillus caldus. A. thiooxidans *shares the same conserved context including a predicted Fur box. In *A. caldus *the OprB family porin-like protein *FeoP *is not contiguous with the *feoABC *cluster but its upstream predicted Fur box suggests its involvement in iron management (Figure 1A; Additional files 1 and 2).

Many organisms have secondary Fe(II) transporters whose primary function is the uptake of other divalent metals such as Mn(II), Mg(II) and Zn(II), but that can, in certain circumstances, also import Fe(II). These include the Nramp-like transporters, such as MntH [21], metal-ABC permeases, such as SitABCD [22] and the ZRT- and IRT-like proteins represented in bacteria by ZupT [23]. However, only potential genes with similarity to *mntH*, a proton-dependent high affinity manganese uptake permease, could be detected in the three Acidithiobacilli. Alignment of the predicted amino acid sequences of MntH demonstrates that it is conserved between *A. ferrooxidans*, *A. thiooxidans *and *A. caldus *and analysis of the upstream DNA predicts the occurrence of a conserved σ_70_-like promoter that overlaps a Fur box in *A. thiooxidans *and *A. caldus *that align with an experimentally validated Fur box in *A. ferrooxidans *(Figure 1B, Additional file 3) [20]. This suggests that *mntH *in the three Acidithiobacilli is regulated by Fur, as has been demonstrated in other bacteria [24-26] and is thus likely to participate in iron transport. This prediction awaits experimental validation.

Genes potentially encoding the FeoPABC and MntH systems were not detected in the Leptospirilla raising the question as to how they assimilate Fe(II). One possibility is that Fe(II) uptake is accomplished by a predicted Ftr1-like permease that is absent in the Acidithiobacilli (Figure 1C). Ftr1 permeases have been shown to operate as iron importers in conjunction with Fet3p-like multicopper oxidases (MCO) in other organisms [27]. Fet3P is a membrane glycoprotein that efficiently oxidizes Fe(II) to Fe(III) for the subsequent transport of Fe(III) into the cytoplasm via Ftr1p [27]. Thus, in the strict sense, these transporters are Fe(III) permeases. In *Leptospirillum *sp. group III, the Ftr1 permease ortholog, named EfeU, is associated with a hypothetical cupredoxin that exhibits amino acid similarity (56%) to subunit II from heme/copper-type cytochrome/quinol oxidase from *Burkholderia pseudomallei *(ABN85317). In *Leptospirillum *sp. group II – UBA this gene pair (EAY57254- EAY57255) is well conserved and linked to a gene encoding a hypothetical protein with a kelch domain (EAY57256). The function of this hypothetical gene is unknown, but kelch motifs are present in other oxidases such as galactose oxidase (pfam01344). These two other functions with oxidase-type motifs could thus be potentially involved in the oxidation of Fe(II) for dedicated EfeU Fe(III) uptake. Lack of (known) Fe(II) transporters in this group of strict iron-oxidizers could be a mechanism to evade iron stress imposed by readily available Fe(II) ions at pH 1. Models for the uptake of Fe(II) for the Acidithiobacilli and Leptospirilla are shown in Figure 1D.

### Ferric Iron Transporter Profiles

**Figure 2:**
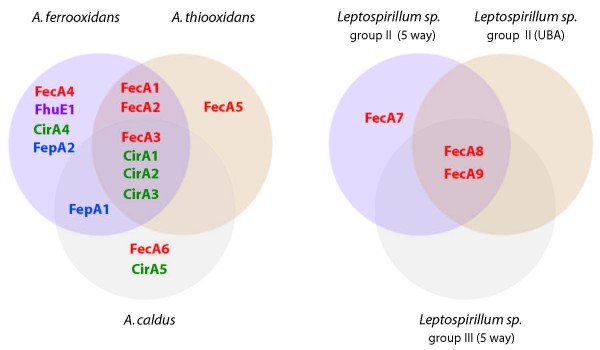
**Abundance and diversity of predicted ferric iron siderophore transporters in Acidithiobacilli and Leptospirilla.** The Ven diagram shows species-specific and shared TonB dependent outer membrane receptors. Color coding indicates predicted siderophore specificity. Red: dicitrate, Green: linear catecholate, Blue: cyclic catecholate, Purple: hydroxamate.

Despite the abundant supply of soluble iron in their low pH biotope, the Acidithiobacilli exhibit a plethora of predicted TonB-dependent Fe(III) transport systems (OMRs). Fourteen different TonB-dependent outer membrane Fe(III) siderophore transporter groups belonging to the FecA-dicitrate, FhuA-hydroxymate, CirA-linear catecholate and FepA-cyclic catecholate type siderophore receptors were predicted, of which 11 are present in *A. ferrooxidans*, 8 in *A. thiooxidans *and 7 in *A. caldus *(Figure 2, Additional file 1). In contrast, the Leptospirilla contain only 3 predicted TonB-dependent outer membrane Fe(III) siderophore receptors, all corresponding to the FecA type of dicitrate transporters (Figure 2).

**Figure 3:**
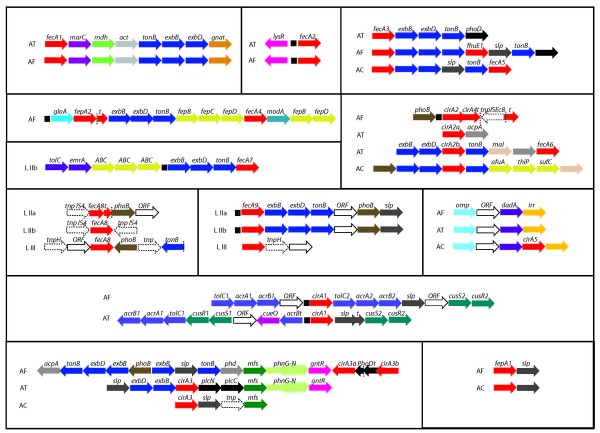
**Genomic context for ferric iron transport candidate genes.** Predicted functions of the genes are listed in Additional file 2 according to gene cluster number. AT: *A. thiooxidans*. AF: *A. ferrooxidans*. AC: *A. caldus*. LIIa: *Leptospirillum *sp. Group II UBA. LIIb: *Leptospirillum *sp. Group II 5-way GC. LIII: *Leptospirillum *sp. Group III 5-way GC.

Four of the predicted transporters are found in all members of the Acidithiobacilli but are only rarely detected in other organisms (Additional file 4). These correspond to the FecA (FecA3) and CirA (CirA1, 2, 3) types, and are conserved in amino acid sequence and gene context within the Acidithiobacilli (Figure 3). For example, FecA3 exhibits 41–43% sequence similarity only to TonB dependent receptors in the α-proteobacteria *Zymomonas mobilis *ZM4 and *Gluconobacter oxydans*. Also, the three predicted CirA receptors exhibit similarity only to those found in the α-proteobacterium *Acidiphilium cryptum*, with which they share the same habitat. This suggests that there is a core group of Fe(III) transporters that are found in the Acidithiobacilli and environmentally related microorganisms that may reflect the specialized needs of these microorganisms for Fe(III) uptake at low pH. The remaining receptors shared only by *A. ferrooxidans *and *A. thiooxidans *(FecA1 and FecA2) or by *A. ferrooxidans *and *A. caldus *(FepA1) and the additional 7 receptors that are found uniquely in only one or other of the Acidithiobacilli exhibit very low similarity (30–40%) with known TonB dependent receptors indicating that they may also be specific for the Acidithiobacilli and could be used for specialized iron uptake requirements in each member of the group (Additional file 4).

The diversity of OMRs is reflected not only in their amino acid sequence diversity, but also in their predicted siderophore affinities and isoelectric points (Figure 2, Additional file 5). The OMRs of the three Acidithiobacilli span a wide range of predicted pIs from 5.57 to 9.15, contrary to other environmentally restricted microbes including the Leptospirilla whose pIs vary in very narrow range from 5.10 to 5.73. This observation raises intriguing questions regarding potential alternative life styles of the Acidithiobacilli as discussed below.

In contrast to the extensive repertoire of Fe(III) receptors exhibited by the Acidithiobacilli, Leptospirilla have only predicted dicitrate-type siderophore receptors, two of which, FecA8 and FecA9, are found in both *Leptospirillum *sp. group II and *Leptospirillum *sp. group III (Figure 3). Recent analysis of an environmental transcriptome suggests that *L. ferrooxidans *may also have a FecA type outer membrane receptor [28]. This paucity of siderophore receptors could be explained by the presence and conservation in all Leptospirilla of an EfeU-type iron transporter. The latter may suffice for the acquisition of soluble Fe(III) at low pH eliminating the need for high affinity iron chelating compounds and/or cognate receptors.

**Figure 4:**
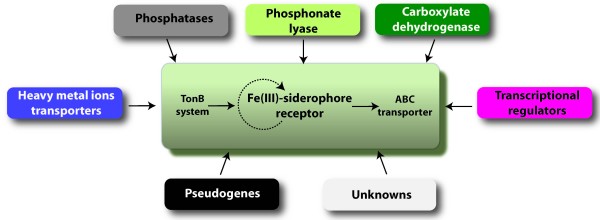
Examples of predicted novel metabolic functions grouped together with iron uptake functions in putative co-regulated gene clusters (operons).

Figure 3 illustrates the organization of the gene clusters in the Acidithiobacilli and Leptospirilla encoding predicted TonB-dependent Fe(III) siderophore OMRs and the presence of predicted Fur boxes. Whereas in most bacteria, genes encoding ferric siderophore outer membrane receptors are clustered and expressed with ABC transporters and TonB systems, the Acidithiobacilli and Leptospirilla appear to have a different organization. Both families exhibit a variety of other genes linked to the transporters including predicted genes for enzymes, transportation and regulatory functions and unknown function (Figure 4). Genetic linkage of these genes with the iron uptake genes argues in favor of a conjunct function and their identification now opens the door for experimental investigation.

### Context-Based Functional Associations Inferences for Iron Uptake

A comparative genomic analysis of gene clusters containing TonB-dependent Fe(III) transport systems was undertaken in order to identify additional genes within the clusters that could potentially be associated with iron uptake. This analysis suggests novel examples of predicted co-regulation of iron uptake functions with genes implicated in (i) citrate biosynthesis and (ii) phosphate metabolism.

#### (i) Citrate biosynthesis

**Figure 5:**
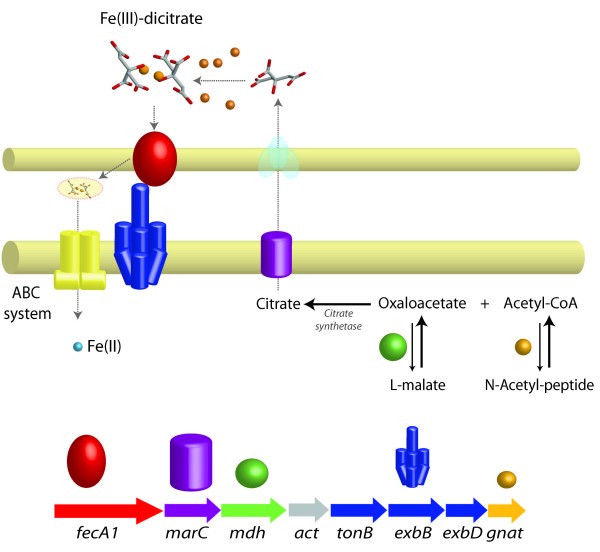
**Predicted novel citrate synthesis-efflux system and Fe(III)-dicitrate uptake system in *A. ferrooxidans *and *A. thiooxidans*.** Inset: Predicted conserved gene cluster coding for a dicitrate TonB-dependent receptor (FecA1), a dicarboxylate efflux pump (MarC), a malate dehydrogenase (Mdh), an ACT domain carrying protein (Act), TonBExbBD biopolymer transport system, and a GNAT acetyltransferase (Gnat). Colors in the membrane model correspond to genes in the gene context scheme.

A gene cluster was identified in *A. ferrooxidans *and *A. thiooxidans *that links a classic dicitrate TonB-dependent Fe(III) uptake system (FecA1) with four genes that we hypothesize encode a novel citrate synthesis and efflux system (Figure 5) including: (i) an efflux pump of the dicarboxylate family that we suggest could serve as a citrate efflux pump – members of this family are known to export small organic molecules [29], (ii) a malate dehydrogenase (family of NAD-dependent 2-hydroxycarboxylate dehydrogenases) that reversibly converts malate to oxaloacetate (an intermediate in the biosynthesis of citrate) [30], (iii) a protein of unknown function that exhibits an ACT domain (pfam01842) typically present in allosteric enzymes with complex regulation involving the binding of ligands [31] and (iv) a predicted acetyltransferase of the GNAT type superfamily members of which use acyl-CoAs to acylate their cognate substrates [32]; this could catalyze the formation of citrate via the acetylation of oxaloacetate by acetyl coenzyme A (Figure 5). This exact gene context has not been detected in other organisms but, in *Bordetella mobilis *and *B. parapertusis*, a predicted dicarboxylate efflux transporter gene is located in a cluster with the FauA receptor for ferric coprogen and ferric-rhodotorulic acid and the alcaligin siderophore synthase [33] resembling the predicted gene organization of the Acidithiobacilli gene cluster.

#### ii) Phosphate metabolism

**Figure 6:**
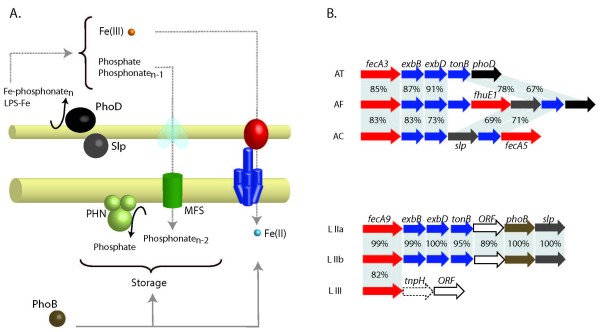
**Model for phosphate/phosphonate associated Fe(III) uptake in Acidithiobacilli and Leptospirilla.** (A) Partially conserved gene cluster in all three Acidithiobacilli coding for a FecA3 TonB-dependent receptor, the biopolymer transport system ExbBDTonB, a surface layer protein (SLP) and an alkaline phosphatase (PhoD). (B) Partially conserved gene cluster in all three Leptospirilla coding for orthologous TonB-dependent receptors (FecA9), the biopolymer transport system ExbBDTonB, a phosphate activated transcriptional regulator (PhoB) and a surface layer protein (SLP). Colors in the membrane model correspond to genes in the gene context scheme. AT: *A. thiooxidans*. AF: *A. ferrooxidans*. AC: *A. caldus*. LIIa: *Leptospirillum *sp. Group II UBA. LIIb: *Leptospirillum *sp. Group II 5-way GC. LIII: *Leptospirillum *sp. Group III 5-way GC.

Four TonB-dependent Fe(III) transport systems in Acidithiobacilli (FhuE1, FecA3, CirA2 and CirA3) and one in Leptospirilla (FecA9) are found in clusters associated with genes predicted to be involved in phosphate metabolism (Figures 3 and 6). These genes include acid and alkaline phosphatases AcpA and PhoD that hydrolyse phosphoester bonds, the membrane associated carbon-phosphorus lyase complex PhnG-M that participates in the degradation of phosphonates and a phosphate/phosphonate transporter of the major facilitator superfamily, the phosphorus regulon regulator PhoB and surface layer proteins of unknown function. These genes are associated with others predicted to be involved in iron uptake and to be regulated by Fur (Figure 3, Additional file 3).

The predicted acid phosphoesterases in the acidophiles belong to a superfamily of bacterial extracellular enzymes that includes phospholipases C, acid phosphatases and alkaline phosphatases of the PhoD family. Alkaline and acid phosphatases are broad substrate specificity or polyspecific enzymes that liberate inorganic phosphate from a range of organic molecules and are well-conserved members of the Pho regulon found in many bacteria [34]. Most C-type phospholipases are membrane active enzymes that hydrolyze both phosphatidylcholine and sphingomyelin and play important roles in disease in a variety of microbial pathogens (e. g in *Pseudomonas aeruginosa *[35] and *Mycobacterium tuberculosis *[36]). Acid phosphatases affect host signaling pathways by dephosphorylation of host proteins and thus interfere with phagosome formation [37] and respiratory burst [38]. The natural substrate(s) of PhoD is still not known [39,40] and little information is available regarding the role of these phosphatases in non-pathogenic bacteria. In addition to the roles described above, these enzymes liberate inorganic phosphate from a range of organic molecules and might enable bacteria to assimilate phosphate from organo- and metallo-phosphates in the environment. Given the gene association profiles detected in the current work and since phosphate is a well known chelator of iron species we hypothesize that phosphate produced by the repertoire of tightly linked phosphatases might function in these acidophiles as an inorganic ferric iron chelator.

Both phosphate and iron uptake related functions in Acidithiobacilli and Leptospirilla are located downstream of OmpR family transcriptional regulators similar to PhoB (Figure 6). Co-localization of iron uptake functions with phosphate metabolism genes and the phosphate responsive transcriptional regulator PhoB, suggests the existence of an iron-phosphate coordinated regulatory control circuit. Coherent regulation of target genes by the iron responsive transcriptional regulator Fur [41] and the phosphate dependent transcriptional activator PhoB [34] could coordinate environmental and intracellular signals for homeostatic gene expression of uptake and storage functions in response to phosphate and/or iron availability. Supporting this argument, one regulator of the PhoB family has been confirmed experimentally to be under Fur control in *A. ferrooxidans *[18]. In addition, transcription profiling data obtained for a *fur *knockout strain of the dissimilatory metal-reducing bacterium *Shewanella oneidensis *MR-1, revealed that *phoB *was repressed in the *fur *mutant [42] further extending the connections in this circuit. Understanding how regulatory factors other than Fur control expression of iron uptake genes is still limited, yet the picture is growing increasingly complex with the recent findings of superimposed positive regulation by several different transcriptional regulators [43]; PhoB could be added to this list.

### Comparative Genomics Based Identification of a Genomic Island Associated with Iron Metabolism

**Figure 7:**
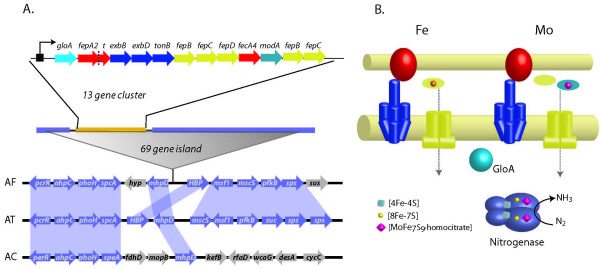
**Nitrogenase dedicated ferric iron and molybdate transport in *A. ferrooxidans*.** A. Genomic context and gene organization of the predicted bifunctional Fe and Mo transport operon. B. Model for *A. ferrooxidans *dedicated metal import for nitrogenase function. Colors in the membrane model correspond to genes in the gene context scheme. ■: Fur box. Violet: Genes encoding nitrogenases. Orange : genes encoding bifunctional metal transporters. AT: *A. thiooxidans*. AF: *A. ferrooxidans*. AC: *A. caldus*.

Taxonomically restricted genes are of special interest because they are expected to play a role in defining exclusive ecological adaptations to particular niches [44]. We predict a genomic island containing a gene cluster associated with iron metabolism in *A. ferrooxidans *that may be an exclusive system of physiological/ecological significance for the bioleaching consortia. The FepA2-FecA4 TonB-dependent Fe(III) transport system comprises a 13 gene cluster (Figure 7) that resides within a predicted genomic island containing 69 genes that is absent from the genomes of *A. thiooxidans *and *A. caldus*. The predicted protein products encoded by the 13 gene cluster include two OMRs with different predicted siderophore affinity, a TonB system and two contiguous partially complete high affinity metal ABC transporter systems (Figure 7, Additional file 1). These two ABC transporters include three high affinity periplasmic solute-binding proteins that differ in size, sequence and ligand specificity (Figure 7). Two of these have predicted affinity for Fe(III) siderophores and one for molybdate. The most similar orthologs to the Mo-binding protein of *A. ferrooxidans *are found in several nitrogen-fixing bacteria. Interestingly, nitrogen fixation is performed by an enzymatic complex made up of a Fe/Mo-protein (the dinitrogenase) and a Fe-protein (the dinitrogenase reductase) [45]. The association of a gene predicted to encode ModA-like periplasmic binding protein with affinity for molybdate with genes predicted to be involved in siderophore uptake suggests that the gene cluster might be a bifunctional ABC transporter system, destined to cover the requirements of both Fe and Mo essential metabolic cofactors.

Adjacent to the proposed 13 gene Fe-Mo transport cluster is a region of 44 genes predicted to be involved in nitrogen fixation including the full set of *nif *genes required for nitrogenase assembly and maturation [13]. Associated with the Fe-Mo transport cluster is a predicted *fixABCX *gene cluster that, in other nitrogen fixing microorganisms, has been shown to be required for electron transfer during nitrogen fixation [46] (Figure 7). These observations suggest that the Fe-Mo transport cluster is the cognate metal transporter for the Mo-Fe nitrogenase.

A gene (*gloA*), predicted to encode a truncated globin, forms part of the 13 gene Fe-Mo transport cluster. Truncated globins have been described in prokaryotes, protozoa, eukaryotic algae and in plants [47]. Their function remains unclear, although they have been shown in plants to exhibit low oxygen affinity and, since their expression is decreased by hypoxia, it has been suggested that their role is to bind oxygen in conditions of high oxygen availability [47]. We hypothesize that the role of *gloA *in *A. ferrooxidans *is to sense oxygen and regulate the transcription of the Fe-Mo transport operon promoting the importation of the required metallic cofactors under conditions suitable for nitrogen fixation.

The absence of both the Fe-Mo transporter cluster and the surrounding nitrogen fixing genes in *A. thiooxidans *and *A. caldus *suggest that this whole region might be a genomic island acquired by lateral gene transfer, as has been suggested for some iron transporting gene clusters [e.g. [48]] and other traits influencing survival, fitness and adaptation in bacteria [49]. An analysis of this region using G+C content analysis, pentanucleotide frequency assessment and codon usage patterns demonstrate that it conforms to the average characteristics of the host genome, however the presence of phage remnants, a site specific tyrosine recombinase and a Val-tRNA adjacent to the region support the contention that it is a genomic island.

### Iron Efflux

Concentration-dependent toxicity of metals that are essential micronutrients can be ameliorated by balancing metal influx and efflux through the use of several different types of efflux pumps (e.g. ATPases, RND, MSF, etc) and metal responsive regulators [8]. Only proteins of the cation diffusion facilitator family (CDF) have been shown thus far to remove iron when present in excess. Two proteins FieF and MamB, have been implicated in this role in *E. coli *[50] and *Magnetospirillum gryphiswaldense *[51] respectively. Six different orthologs of FieF and/or MamB were predicted in the Acidithiobacilli (termed CdfA-C) and the Leptospirilla (termed CdfD-F) (Additional file 6). Their role in iron efflux is suggested by the conservation of functional motifs typical of the FieF protein subfamily (PRK09509). This hypothesis is strengthened by gene context analysis in other microorganisms, which reveals frequent juxtaposition of CDFs with iron related functions, such as: the iron uptake regulator Fur, the iron detoxification protein Dps, Fe/Pb or Fe/Zn permeases, the ferri-siderophore receptor FecA, Fe-S cluster assembly proteins and Fe-Mo cofactor proteins. For example, one of the predicted cation diffusion facilitator family proteins is encoded within a three gene cluster immediately adjacent to the Fe-Mo uptake transporter from *A. ferrooxidans *described above. This cluster consists of a predicted ABC solute binding protein, an RND-type outer membrane factor similar to OprD and the cation diffusion facilitator CdfB. The ABC solute binding protein exhibits 70% similarity to the sulfate/molybdate binding protein ModA (COG0725) and weak similarity (less than 30%) to the ABC-type Fe(III) binding protein AfuA (COG1840), while CdfB carries a C-terminal signature (MTH1175) found in several uncharacterized proteins belonging to the Fe-Mo cluster binding proteins. This suggests a role for this gene cluster in Mo or Fe efflux. Consistent with this hypothesis is the fact that this cluster is contiguous with a predicted bifunctional uptake system for these two ions and with several gene clusters encoding the Fe-Mo nitrogenase.

### Iron Storage

**Figure 8:**
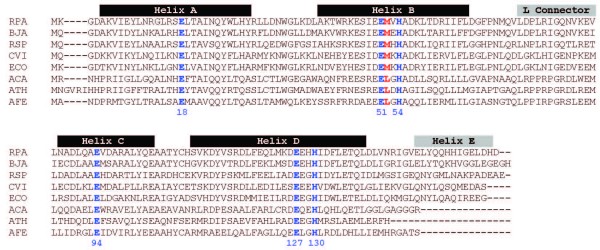
**Sequence alignment of bacterioferritins. ***Rhodopseudomonas palustris *(RPA) NP_948938, *Bradyrhizobium japonicum *(BJA) NP_773320, *Rhodobacter sphareoides *(RSP) YP_351589, *Chromobacterium violaceum *(CVI) NP_903069, *E. coli *(ECO) NP_417795, *A. caldus *ACA, *A. thiooxidans *ATH and *A. ferrooxidans *AFE. The binuclear metallic center is indicated in blue (Glu-18 Glu-51 His-54 Glu-94 Glu-127 His-130) and the heme ligand in red (Met-52).

Problems associated with toxicity and low availability of iron can be alleviated in bacteria by the use of iron-storage proteins such as the heme-containing bacterioferritins and the heme-free ferritins [52]. In addition, iron detoxification proteins of the Dps protein family are employed in the protection of DNA from iron-induced free radical damage [52]. Of these three protein types, bacterioferritins are the most ubiquitous in bacteria and they were the only class detected in the Acidithiobacilli. Alignment of their amino acid sequences demonstrates that they are conserved between *A. ferrooxidans*,* A. thiooxidans *and *A. caldus *including all residues implicated in the ferroxidase center (Figure 8). This center endows the protein with the ferrous-iron-oxidizing activity to store iron in its core [52].

Typically, bacterioferritins contain up to 12 protoporphyrin IX heme groups bound symmetrically at the interface of two adjacent subunits by the residues Met52 and Met52' [53]. In all three Acidithiobacilli the equivalent position is replaced by a leucine residue. Interestingly, *E. coli *bacterioferritin mutants modified at Met-52 appear to be correctly assembled and are still capable of accumulating iron, but lack the heme groups involved in mediating iron-core reduction and iron release [54]. This conserved substitution suggests that orthologs of bacterioferritin present in the Acidithiobacilli could: a) use a residue different from Met52 for the coordination of heme moieties, or b) lack heme groups and thus also lack from the capacity to reutilize the iron stored inside its cavity or c) utilize a different mechanism for iron-core reduction and metal release.

Orthologs of the classical iron storage proteins could not be detected in the Leptospirilla, raising the question as to how they store iron or indeed if an iron storage mechanism is required at all, given that they are restricted to living in environments with high soluble iron loads. One possibility is that they can store iron in intracellular polyphosphates inclusions as has been suggested for *E. coli *[55]. Although there are no reports regarding the capacity of the Leptospirilla to accumulate polyphosphate granules, the presence of a predicted polyphosphate kinase 2 and an exopolyphosphatase known in other organisms to be involved in polyphosphate biosynthesis and utilization, suggests that phosphate reserves might contribute to the storage of divalent cations like iron. In the case of *A. ferrooxidans*, it has been shown that the bacterium accumulates substantial numbers of polyphosphate granules (400 nmol of Pi/mg of protein) under Pi sufficient growth conditions [56] raising the possibility that these granules might also store iron. This hypothesis can now be experimentally investigated.

Alternatively, obligatory Fe(II)-oxidizing acidophiles could bypass the absence of storage proteins by making use of their inherent capacity to transform the soluble and life threatening Fe(II) to the less soluble Fe(III). This transformation could serve as a protection mechanism by promoting tightly controlled Fe(III) uptake.

### Iron responsive regulator profiles

Genomic evidence indicates that the Acidithiobacilli and Leptospirilla have a diverse set of transcriptional regulators of the Fur family, corresponding to well known regulators involved in the maintenance of divalent cation homeostasis and the response to several environmental stresses [57]. Within this set, occurrence and conservation of the iron responsive Fur regulator points to conserved regulation mechanisms of the expression of iron related functions.

**Figure 9:**
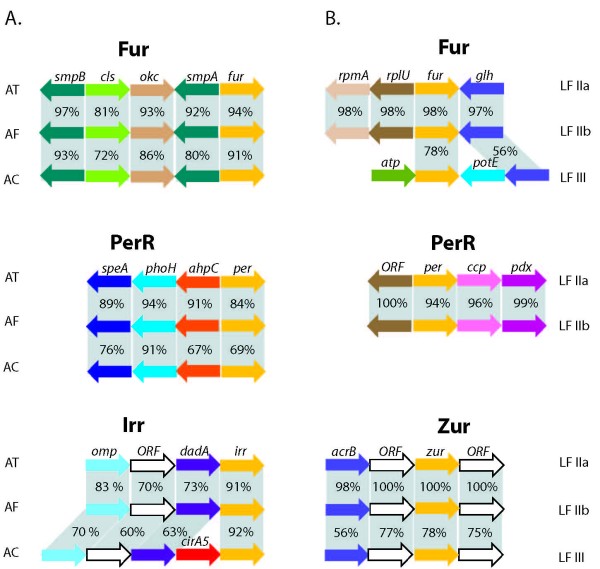
**Fur family transcriptional regulators and their genomic context in A) Acidithiobacilli, B) Leptospirilla.** Color coding indicates orthology and bars linking genes through genomes indicate percentage of amino acid sequence similarity. AT: *A. thiooxidans*. AF: *A. ferrooxidans*. AC: *A. caldus*. LIIa: *Leptospirillum *sp. Group II UBA. LIIb: *Leptospirillum *sp. Group II 5-way GC. LIII: *Leptospirillum *sp. Group III 5-way GC.

Fur from *A. ferrooxidans *has been demonstrated experimentally to be functional [18,20]. In the Acidithiobacilli two other members of the Fur family are predicted: a heme responsive (Irr-type) regulator responsible for the control of heme biosynthesis in response to iron availability and a peroxide responsive (PerR-type) regulator responsible for the control of a variety of basic physiological processes in response to peroxide stress (Figure 9A) [57,58]. Amino acid sequence similarity and gene context conservation between Fur, PerR and Irr from *A. ferrooxidans *and the other two Acidithiobacilli suggest similar regulatory roles in the three bacteria (Figure 9A). In *A. caldus*, one TonB-dependent ferri-siderophore receptor (CirA5) is encoded immediately upstream of the proposed Irr-like Fur family regulator, suggesting a role in iron uptake beyond that of heme biosynthesis as has been reported for several α-proteobacteria [59].

The Leptospirilla are also predicted to encode three members of the Fur family (Figure 9B): an iron responsive Fur-type regulator, a peroxide sensitive PerR-type regulator and a zinc responsive Zur-type regulator [60]. Gene context analysis further supports a role for the PerR-like regulator in alkylperoxide stress response in *Leptospirillum *sp. group II, where a cytochrome c peroxidase and a peroxiredoxin of the AhpC/Tsa family are divergently transcribed. Partial conservation of this context occurs in the Acidithiobacilli, where the gene divergent to PerR is also a peroxiredoxin of the AhpC/Tsa family. Analysis of the genetic context of Fur and Zur in *Leptospirillum *type II provides no additional insights into their functions.

Bioinformatic analysis of predicted Fur binding sites identified thirteen candidate sites in the Acidithiobacilli and Leptospirilla. Three of the predictions in *A. ferrooxidans *correspond to previously documented Fur binding sites. Novel sites were mapped to their respective genomic contexts (Figure 3) and are presented in Additional file 3. The strongest predictions with the information theory motif model occurred in Leptospirilla type II.

Most Fur regulons exhibit overlapping iron uptake functions, and this also seems to be the case in acidophiles. Many bacterial species have extensive and largely conserved Fur regulons, several of which include: a) the *mntH *gene, b) the *feoAB*, and c) one or several TonB-dependent outer membrane receptor genes [e.g. [61]]. Presence of Fur boxes in the promoters of other transcriptional regulators is also not unprecedented and suggests additional regulation of the linked iron uptake functions by positive regulators coordinated with iron availability.

## Discussion

Detailed comparative analysis of the iron management functions in acidophiles shows that:

• *different absolute numbers of iron acquisition systems are present in the acidophiles*. Variations in gene content may reflect adaptive advantages to their respective ecological niches.

• *absence of sensu stricto Fe(II) transporters and paucity of Fe(III) transporters in the Leptospirilla*. This could represent a strategy to evade iron stress imposed by readily available iron at constant low pH.

• *significant diversity of iron uptake functions exists in the Acidithiobacilli*. The diversity of outer membrane receptors exhibited by *A. ferrooxidans*, *A. thiooxidans *and *A. caldus *might be considered an unexpected feature for extreme acidophiles, inhabiting conditions typically rich in soluble iron. This diversity might instead reflect the range of different pH environments (from pH 1 to pH 5) with varying iron bioavailability known to be inhabited by these bacteria.

• *iron functions are predicted to be organized in gene clusters together with several genes encoding non-iron related functions*. These clusters are predicted to be operons and to function in the same pathway or functional module. They might have evolved by stepwise accumulation of coherent functional sub-clusters, for example: a) a citrate biosynthesis and exportation module and a ferric dicitrate uptake module, b) an iron uptake module and a phosphate/phosphonate uptake module.

• *iron storage and iron efflux function are predicted in the Acidithiobacilli*. Presence of a bacterioferritin, polyphosphate accumulation functions and variants of FieF-like diffusion facilitators indicated that the Acidithiobacilli may remove or store iron under conditions of variable availability.

• *capacity to oxide iron could itself be a way to evade iron stress*. Fe(II)-oxidizing acidophiles transform the soluble and life threatening Fe(II) to the less soluble Fe(III). This could serve as a protection mechanism via the co-precipitation of Fe(III) with sulfates and phosphates, not only for themselves but also for other microorganisms co-inhabiting the same ecological niche.

**Figure 10:**
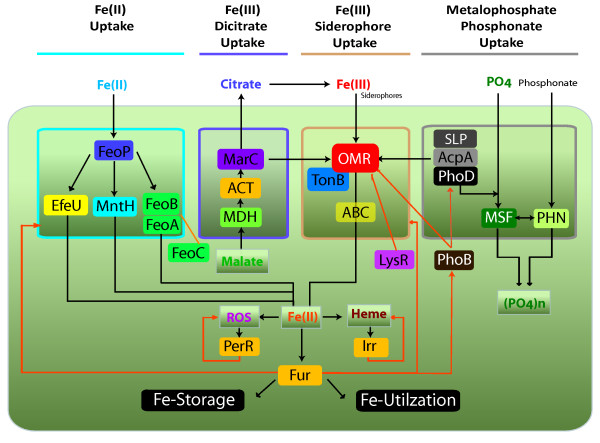
**Diversity of alternative iron acquisition modules and putative regulatory connections in acidophiles.** Light blue: ferrous iron uptake module, Violet: Ferric-dicitrate uptake module, Orange: Ferric-siderophore uptake module, Grey: Metalophosphate/phosphonate uptake module, Orange arrows: Regulatory connections.

Even when conserved core modules of genes involved in iron-related functions are strongly predicted, such as the OMR-TonB system, associated genes vary from bacterium to bacterium. For example, while both the Leptospirilla and the Acidithiobacilli share a connection between iron uptake genes and phosphate metabolism genes, only *A. ferrooxidans *and *A. thiooxidans *have ferric iron uptake systems linked to citrate production and exportation. These observations highlight the existence of a diversified set of predicted alternative iron acquisition modules. The evidence presented herein clearly indicates that, in this particular group of acidophiles, several redundant or alternative iron transporter modules are present (Figure 10). Their identification helps to interpret the physiology/ecology of these organisms.

The existence of redundant modules is a likely indication that these acidophiles are versatile in iron acquisition-related functions. This versatility could represent an advantage in changing environmental conditions such as might be found in naturally acidic conditions and in industrial copper bioleaching heaps in which differences in iron availability could arise due to variations in the environmental pH, directly affecting iron solubility. In addition, iron biooxidation can at least partially deplete the environment of soluble ferrous iron by oxidizing it to the ferric form that can co-precipitate with other components such as phosphate or sulfate to form insoluble complexes (e.g. jarosite). A ready source of this sulfate comes from associated sulfur oxidizing bacteria. Niche partitions and ecological successions between iron- and sulfur oxidizing acidophilic microorganisms could be at least partially explained by such changes in iron bioavailability.

As had been observed earlier for *A. ferrooxidans *[17], the TonB-dependent outer membrane siderophore receptors of *A. thiooxidans *and *A. caldus *span a wide range of predicted pIs from 5.57 to 9.00 and 6.15 to 8.45, respectively. This evidence, and the diversity of siderophore specificities predicted for these three microorganisms, suggests that the OMRs from the Acidithiobacilli represent functional iron uptake genes working at different pHs and/or taking up different iron (ferri-siderophore) sources at a given pH. In accordance with this hypothesis, different pH conditions of growth of *A. ferrooxidans *in iron (pH 1.6 – 2.0) or sulfur (pH 3.5 – 5.0), which impose different iron availability restraints, have been demonstrated [17] and a broad range of growth from pH 1.0 – pH 4.0 in tetrathionate media has been described for *A. caldus *[62]. At pH values above 2, Fe(III) generated by the biooxidation of Fe(II) has low solubility and starts to precipitate. Furthermore, sulfate produced during sulfur oxidation strongly interacts with Fe(III) and forms complex iron oxides precipitates lowering the concentration of soluble iron [63]. As a consequence, when growth occurs at pHs above 2, the Acidithiobacilli might be compromised by the lack of bioavailable Fe(III) and thereby have a greater need for high affinity Fe(III) transporters of diverse types. In contrast, the Leptospirilla grow in a more acidic and restricted pH range (pH 1 or lower) and do not oxidize sulfur [15]. Thus, their paucity of different outer membrane receptors with a narrow range of isoelectric points (from 5.10 to 5.73) could result from their uncompromised access to a constant source of soluble iron.

We speculate that the observed ecophysiology of the Acidithiobacilli and the Leptospirilla could be partially explained by these differences in genetic determinants for iron uptake. For example, in industrial bioleaching heaps pHs range from 1 to 5 and a significant variation in iron speciation occurs (personal communication, C. Demergasso) perhaps prompting the need for a diverse range of siderophores to mediate iron uptake. These siderophores could be produced by other indigenous microbial species present and subsequently scavenged by the Acidithiobacilli. On the other hand, the abundant soluble iron present in very low pH environments, for example in Iron Mountain [15] or in industrial biooxidation tanks [11], allows Leptospirilla to survive with few TonB iron uptake systems and to become the dominant microorganisms. In addition, in biooxidation tanks there is a build-up of Fe(III) to very high concentrations over time that could also preclude the necessity for multiple TonB uptake systems.

The diversity of OMRs may also have a profound impact on bacterial survival and genomic stability/plasticity for other reasons. Outer membrane receptors are multifunctional proteins involved in the uptake of several structurally and functionally unrelated substances and may serve as receptors for colicins and bacteriophages [64-66]. Sensitivity to these agents may also be TonB dependent [67]. Thus, the gene clusters encoding the Ton system and specific OMRs may deliver phages/colicins into the cell contributing to bacterial survival and competition in the environment. For example, by competing for the same receptor protein, the siderophore ferrichrome inhibits killing of *E. coli *cells by colicin M [68] and by phage phi 80 [69]. Since infection is dependent on the functional state of the receptor protein and the latter is dependent on the energized state of the cell and TonB function, susceptibility to infection differs between actively growing and partially starved cells [70]. Thus, actively growing cells that are proficiently taking up iron can be resistant to phages and colicins delivered by other bacteria, provided that the cognate siderophores are also secreted by some member of the consortia.

Mechanisms that help the microorganisms to deal with changes in iron and siderophores concentrations or phage titres are thus deemed to be critical for fitness and survival of bioleaching microbes and their understanding might contribute to improving the capacity to control the bioleaching processes.

## Conclusion

An analysis of the coding potential, conservation, organization and distribution of iron management functions of acidophilic bacteria is beginning to identify the molecular adaptations that underpin their ability to cope with potentially high concentrations of soluble iron at low pH and varying concentrations at other pHs. Microorganisms of the Acidithiobacilli family, that can grow between pH 1–5 using iron or sulfur (*A. ferrooxidans*) or just sulfur (*A. thiooxidans and A. caldus*) as energy sources, exhibit a surprisingly large number of different predicted iron transporters that potentially allow them to grow in conditions with less abundant iron and to compete for iron with other microorganisms present in their niche. On the other hand, the obligatory iron oxidizing Leptospirilla that thrive only at extremely low pH (pH1) have only a few predicted iron uptake mechanisms. These differences can help explain the distribution and activity of these two groups in naturally acidic environments and in industrial bioleaching operations. These initial findings lay the framework for future work aimed at understanding how iron uptake and homeostasis is regulated in acidophiles, including how iron-oxidizers discriminate between iron as a micronutrient and as an energy source. It also suggests how consortia of microorganisms can operate synergistically or antagonistically to recover minerals during bioleaching and will pave the way for a better understanding of this important biotechnological process.

## Methods

### Sequence Data

The complete genome sequence of *Acidithiobacillus ferrooxidans *ATCC 23270 (AF) was obtained from the Institute for Genomic Research database (TIGR) [13,71]. Draft genome sequences of *Acidithiobacillus thiooxidans *ATCC 19377 (AT) and *Acidithiobacillus caldus *ATCC 51756 (AC) were obtained from the Center for Bioinformatics and Genome Biology (CBGB) [14]. Draft genome sequences of *Leptospirillum *sp. group II UBA (L IIa) and 5 Way GC (L IIb), and *Leptospirillum *sp. group III 5 Way GC (L III) were obtained from the Joint Genome Institute (JGI) [72] and the Genome database from the National Center for Biotechnology Information (NCBI) [73].

### ORF Prediction

ORFs likely to encode proteins were predicted by GLIMMER [74]. This program, based on interpolated Markov models, was trained with ORFs larger than 600 bp from the proper genes available in GenBank and our private databases. All predicted proteins larger than 100 amino acids were searched against a nonredundant protein database as described [75]. Manual curation of the predicted genes was performed to correct errors in start site prediction and identify missing candidate genes. The 5' and 3' regions of each ORF were inspected to define initiation codons using homologies, position of ribosomal binding sites, and transcriptional terminators.

### Gene Identification

The following bioinformatic programs were used to further characterize candidate genes and their predicted protein products: BlastP and PsiBlast [76], the suite of protein characterization programs available in InterproScan [77], Blocks [78] and ClustalW [79]. COGs [80] and two sets of Hidden Markov Models were used to determine ORF membership in families and superfamilies: PFAM V5.5 [81] and TIGRFAMS 1.0 H [82]. The annotated genomes were displayed in the interactive format of Artemis [83]. Genes were deposited in GenBank under the accession numbers: ACI62867–ACI62983 and FJ410133–FJ410136.

### Fur box Identification

A set of 66 experimentally confirmed Fur boxes from *E. coli*, *Salmonella typhimurium*, *P. aeruginosa *and *Staphylococcus aureus *was used to generate an alignment matrix and a weight matrix by the information content method [84]. The weight matrix used to search the all complete and partial genomic sequences included in this study using a 19-bp sliding window as described previously [18]. Genes carrying candidate Fur boxes in their upstream regions were retained as putative iron related functions directly targeted by the Fur regulator and further used as search queries.

### Comparative Genomics

A comprehensive search in the NCBI public database was performed to identify all proteins that are related to iron homeostasis in bacteria using textmining strategies. Amino acid sequences for the iron homeostasis related genes identified were then searched in the genome sequence of *A. ferrooxidans *and draft genome sequences of *A. caldus*, *A. thiooxidans *and the Leptospirilla using wu-BLAST [85] and candidate genes were then compared against each other. Orthologous and paralogous families were derived by performing all-versus-all searches on the remaining protein sequences by using a modified version of a previously described method. Pentanucleotide frequency assessment and codon usage patterns were performed following the criteria established by Merkl [86].

## Authors' contributions

HO carried out the gene identification and analysis for the Acidithiobacilli. VM carried out the gene identification and analysis for the Leptospirilla. PAN carried out the comparative genomic analysis for iron storage and iron efflux. DSH and RQ conceived the study, helped in the biological interpretation, and drafted the manuscript. All authors read and approved the final manuscript.

## Supplementary Material

Additional file 1 **Gene identification table**. Gene annotations for main components of the iron management response in Acidithiobacilli and Leptospirilla. Click here for file

Additional file 2 **Genomic context table**. Gene annotations for Fe(II) uptake, Fe(III) uptake and iron regulation gene clusters. Click here for file

Additional file 3 **Predicted Fur binding sites table**. Fur box sequences, scores and distances to the translation start sites of candidate Fur regulated genes. IT: Information theory, PF: Phylogenetic footprinting, HMM: Hidden Markov Models. Click here for file

Additional file 4 **Best hits for OMRs in GenBank database**. Occurrence, E-values and accession numbers for Best Hits in GenBank for the Acidithiobacilli and Leptospirilla TonB-dependent OMRs. Click here for file

Additional file 5 **Predicted isoelectric points for TonB-dependent outer membrane receptors**. Signal peptides, domains and predicted isoelectric points for TonB-dependent OMRs from Acidithiobacilli and Leptospirilla. Click here for file

Additional file 6 **Predicted iron efflux functions table**. Gene annotations, motifs and genomic context for best hits for predicted iron efflux functions from Acidithiobacilli and Leptospirilla. Click here for file
